# Time series analysis of dengue fever and weather in Guangzhou, China

**DOI:** 10.1186/1471-2458-9-395

**Published:** 2009-10-27

**Authors:** Liang Lu, Hualiang Lin, Linwei Tian, Weizhong Yang, Jimin Sun, Qiyong Liu

**Affiliations:** 1 National Institute for Communicable Disease Control and Prevention, China CDC, Beijing, PR China; 2 Stanley Ho Centre for Emerging Infectious Diseases, School of Public Health, Chinese University of Hong Kong, Hong Kong SAR, PR China; 3 Chinese Center for Disease Control and Prevention, Beijing, PR China; 4 Zhejiang Provincial Center for Disease Control and Prevention, Hangzhou, PR China; 5 State Key Laboratory for Infectious Disease Prevention and Control, Beijing, PR China

## Abstract

**Background:**

Monitoring and predicting dengue incidence facilitates early public health responses to minimize morbidity and mortality. Weather variables are potential predictors of dengue incidence. This study explored the impact of weather variability on the transmission of dengue fever in the subtropical city of Guangzhou, China.

**Methods:**

Time series Poisson regression analysis was performed using data on monthly weather variables and monthly notified cases of dengue fever in Guangzhou, China for the period of 2001-2006. Estimates of the Poisson model parameters was implemented using the Generalized Estimating Equation (GEE) approach; the quasi-likelihood based information criterion (QICu) was used to select the most parsimonious model.

**Results:**

Two best fitting models, with the smallest QICu values, are selected to characterize the relationship between monthly dengue incidence and weather variables. Minimum temperature and wind velocity are significant predictors of dengue incidence. Further inclusion of minimum humidity in the model provides a better fit.

**Conclusion:**

Minimum temperature and minimum humidity, at a lag of one month, are positively associated with dengue incidence in the subtropical city of Guangzhou, China. Wind velocity is inversely associated with dengue incidence of the same month. These findings should be considered in the prediction of future patterns of dengue transmission.

## Background

Dengue fever is an arboviral disease transmitted by mosquitoes in tropical and subtropical areas around the world. It is caused by one of four closely related but antigenically distinct virus serotypes (DEN-1, DEN-2, DEN-3, and DEN-4) of the genus *Flavivirus *[1]. In terms of the number of human infections occurring globally, dengue fever is considered to be the most important arthropod-borne viral disease in humans; currently, more than 2.5 billion people live in high risk areas of dengue fever [2].

In China, outbreaks of dengue-like illness were not uncommon in the 1940s although etiological and epidemiological investigations were not carried out. Outbreaks of dengue-like illness were not reported in China during 1950-1978. An outbreak of dengue fever due to DEN-4 virus occurred in Foshan City of Guangdong Province in 1978 [3]. Since then, dengue occurred frequently in southern China, including Guangdong, Guangxi, Hainan, Taiwan, Fujian and Zhejiang. The dengue fever epidemics in China were caused by all four types of dengue virus [3]. *Aedes aegypti *was the primary vector in the coastal areas of the tropical zone below 22° north latitude, whereas *Aedes albopictus *has a vast area of distribution in China, from 41° north latitude to the southern reaches of the country [4]. As reviewed by Gratz [5], *Aedes albopictus *is considered the primary vector in parts of China where *Aedes aegypti *is uncommon, and a secondary or maintenance vector in other areas. Given the widespread distribution of competent vectors, global warming, and the increasing population movement, dengue is likely to be a continuous threat in China for many years to come [4].

Monitoring and predicting dengue incidence facilitates early public health responses to minimize morbidity and mortality. Weather variables (including temperature, rainfall, and humidity, etc) as potential predictors of dengue incidence have been examined in time series studies [6-9]. Few studies are available, however, on the effect of weather variables on dengue transmission in Guangzhou City of China, where frequent outbreaks of dengue occurred in recent years [10]. In the current study, we used the time-series regression approach to examine the effect of weather variability on the incidence of dengue fever in the subtropical city of Guangzhou for the period of 2001-2006.

## Methods

**Figure 1:**
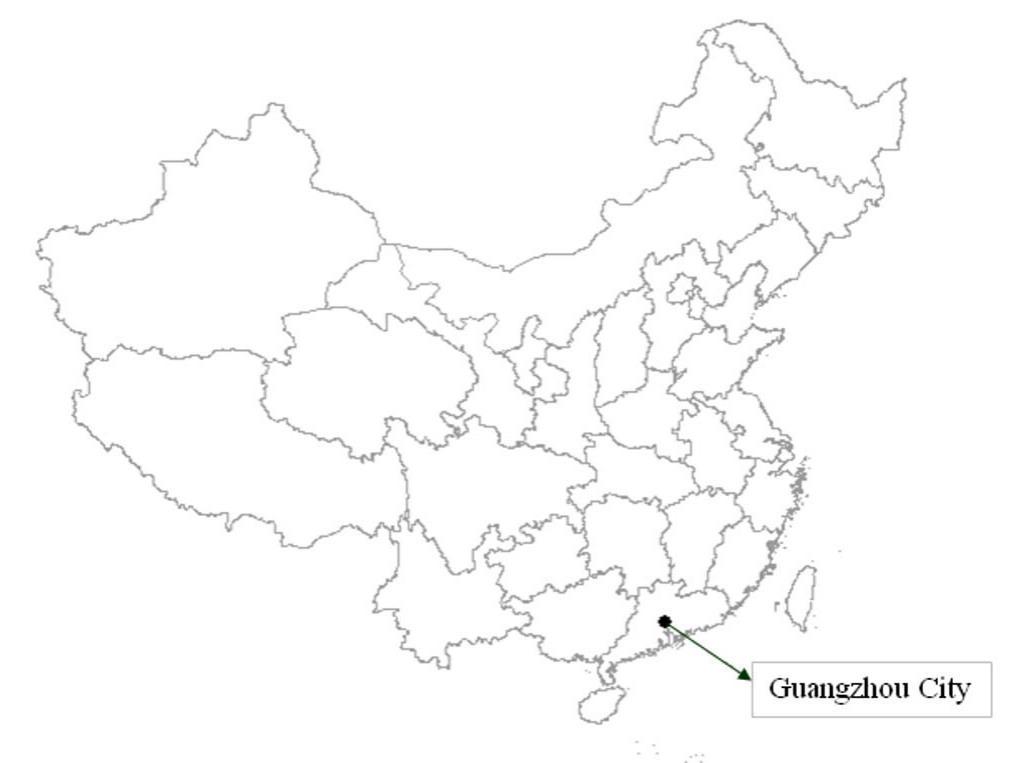
**Location of Guangzhou in China**.

Guangzhou is the capital city of Guangdong Province in southern China. It is located at 112°57'E to 114°3'E and 22°26'N to 23°56'N (Figure 1). The population in 2006 was about 10 million in the metropolitan area. Guangzhou has a humid subtropical climate influenced by the Asian monsoon. Summers are wet with high temperatures and a high humidity index. Winters are mild, dry and sunny. The annual mean temperature ranges from 18°C to 25°C. The annual rainfall is typically between 1,500 mm and 2,000 mm.

Dengue fever is a legally notifiable infectious disease in China. Monthly notified dengue fever cases in the metropolitan area of Guangzhou City from 2001 to 2006 were retrieved from the Notifiable Infectious Disease Report System in China Centre for Disease Control and Prevention (China CDC). Monthly weather data, including minimum temperature (T*min*), maximum temperature (T*max*), total rainfall, minimum relative humidity (H*min*) and wind velocity, were retrieved from China Meteorological Data Sharing Service System for the years 2001-2006.

We performed Spearman rank correlation tests to examine the relationship between monthly dengue incidence and weather variables with a lag of zero to three months. The monthly dengue incidence was modeled using a generalized estimating equations (GEE) approach, with a Poisson distribution. This model enables both specification of an over-dispersion term and a first-order autoregressive structure that accounts for the autocorrelation of monthly numbers of dengue cases. A basic multivariate Poisson regression model can be written as:



The model that adjusts for first-order autocorrelation can be written as:



where T*min*, T*max*, Rain, Wind and H*min *stand for monthly minimum and maximum temperatures, total rainfall, minimum relative humidity and wind velocity, respectively.

As GEE are not a full likelihood-modeling method, the Akaike information criterion (AIC) cannot be used for model selection. We therefore computed the quasi-likelihood based information criterion (QICu) developed by Pan [11] to select the most parsimonious model. Highly correlated explanatory variables were included in separate models to avoid multicollinearity. When using QICu to compare two models, the model with the smaller statistic was preferred. We considered models with ΔQICu ≤ 2 to be equivalent and preferred the model with fewest parameters. All analyses were performed using SAS version 9 for Windows (SAS Institute, Inc., Cary, North Carolina).

## Results

**Figure 2:**
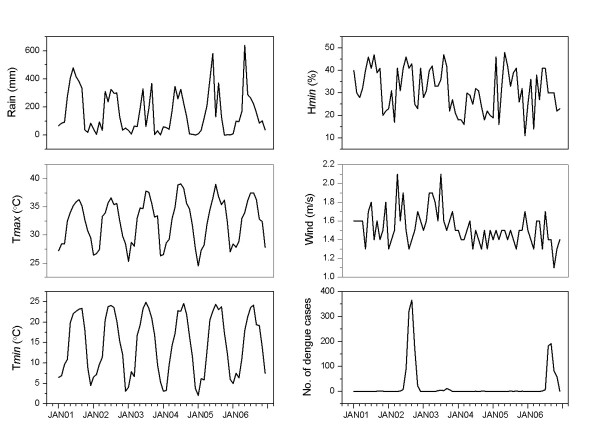
**Monthly weather measurements and the number of dengue fever cases in the city of Guangzhou, China, for the period 2001-2006**.

Figure 2 shows the monthly number of dengue fever cases and weather measurements in the city of Guangzhou, China, for the period 2001-2006. A total of 1,549 cases were reported over the study period. The case numbers were the highest in 2002 and 2006. A seasonal pattern was apparent with most cases occurring from June to November. Seasonal variations in the rainfall and temperature variables were characterized by the highest measurements from April to October. Relatively larger inter-annual variations were observed for the minimum humidity and wind velocity measurements. Two valleys in wind velocity corresponded to the two peaks of dengue cases in 2002 and 2006, respectively.

Table 1 shows the Spearman correlation analysis for the relationship between dengue incidence (2001-2006) and weather variables with a lag of zero to three months. Monthly minimum and maximum temperatures, total rainfall and minimum relative humidity, at lags of zero to three months, were positively associated with the monthly dengue fever cases in Guangzhou over the study period. Monthly wind velocity was inversely correlated with dengue incidence at lags of zero and one, though no statistical significance was observed.

**Table 1:** Coefficients of Spearman correlation analysis between dengue fever cases (2001-2006) and weather variables in Guangzhou, China (* p < 0.05)

**Lag months**	**T*min***	**T*max***	**Rain**	**Wind**	**H*min***
0	0.551*	0.456*	0.179	-0.125	0.188
1	0.665*	0.629*	0.461*	-0.100	0.379*
2	0.656*	0.597*	0.494*	0.155	0.466*
3	0.437*	0.381*	0.441*	0.182	0.326*

Table 2 presents the two best fitting models, with the smallest QICu values, to characterize the relationship between monthly dengue incidence and weather variables. Compared with Model I, the addition of minimum humidity in Model II provided a better fit (ΔQICu = 6.8) although its positive effect on dengue incidence did not reach a level of statistical significance (*β *= 0.058, *p *= 0.113). The number of dengue cases was first-order autoregressive as the number of notified cases of dengue in the current month was related to that in the previous month (*β *= 0.008, *p *< 0.0001). Minimum temperature at a lag of one month had a positive effect on dengue incidence (*β *= 0.317, *p *< 0.0001); wind velocity had a negative effect on dengue incidence of the same month (*β *= -0.465, *p *= 0.0004). In summary, minimum temperature at a lag of one month and wind velocity of the same month were found to be important predicting variables for the monthly dengue incidence for the period of 2001-2006 in Guangzhou, China.

**Table 2:** Time-series Poisson regression of the monthly dengue cases (2001-2006) on the weather factors in Guangzhou, China

	**Model I**			**Model II**		
		
	***β***	**S.E.**	***p***	***β***	**S.E.**	***p***
Case (lag1)	0.008	0.001	< .0001	0.008	0.001	< .0001
T*min *(lag1)	0.351	0.077	< .0001	0.317	0.071	< .0001
Wind (lag0)	-0.380	0.132	0.004	-0.465	0.132	0.0004
H*min *(lag1)				0.058	0.036	0.113
QICu	-425.4			-432.2		

## Discussion

Time series analysis has been used extensively to study the effect of weather variability on dengue [6-9] and other vector-borne diseases [12-17]. In the current study, we used Poisson regression based on generalized estimating equations (GEE) to examine the effect of weather variability on the incidence of dengue fever in the subtropical city of Guangzhou for the 2001-2006 period. The results indicate that an increase in minimum temperature and a decrease in wind velocity are associated with an increase of dengue incidence. Humidity may also be a precipitating factor for dengue transmission. Rainfall and maximum temperature are positively associated with the number of notified dengue cases while they fail to enter the best fitting predictive models.

Minimum temperature at a lag of one month is positively associated with dengue incidence in the study area. This finding is in general agreement with other studies [7,18] in which minimum temperature is reported to be a precipitating factor for dengue transmission. Temperature affects the potential spread of the dengue virus through each stage in the life cycle of the mosquito. Lower temperatures adversely affect the survival of adult and immature *Aedes *mosquitoes while higher minimum temperatures might assist larvae survival in winter. Temperature also affects the extrinsic incubation period (EIP), the period between when the mosquito imbibes virus-laden blood and actually becomes infectious. The EIP is longer at lower temperatures, which causes the mosquito to be less likely to survive long enough to transmit the virus.

Wind velocity is inversely associated with the dengue incidence of the same month. Similar observation was also reported in Barbados where wind speed at a lag of 3 weeks had a negative effect on dengue transmission [6]. This effect could be attributed to a reduced mosquito density due to higher wind velocity. A negative correlation was found between wind velocity and mosquito density [19]. Wind tends to suppress mosquito flight and thus could have affected their oviposition. Increasing wind velocity generally causes a decrease in mosquito flight with the threshold velocities for flight inhibition in the order of 1-4 m/s [20].

Minimum humidity may also contribute to dengue transmission as its inclusion in the predictive model of dengue incidence provided a better fit. Relative humidity affects the survival of mosquito eggs and adults. Newly laid eggs are subject to desiccation and the adults to moisture-related reductions in survival throughout their lifetimes. Relative humidity is probably a limiting factor for dengue transmission in this subtropical city of Guangzhou.

Although rainfall generally increases the number of breeding places for mosquitoes, it fails to enter the best fitting predictive model of dengue incidence in this study. The breeding sites for dengue vectors are primarily containers such as drums, discarded tires, and leaf axils filled with water either manually or by rainfall. When containers are manually filled for water storage in the urban area, vector abundance can be largely independent of rainfall. In addition, heavy rainfall or storms may destroy existing breeding sites, interrupt the development of mosquito eggs or larvae, or simply flush the eggs or larvae out of the pools.

It should be acknowledged that dengue transmission is very complicated. Along with the weather variables, other environmental and host factors such as community intervention measures and human behaviors also influence mosquito populations and the degree of contact between human beings and vector. As systematic mosquito data were not available in the study area, this study focused only on weather variables and therefore the model from this project is not perfect. Additional studies that incorporate more variables such as density of mosquitoes [21,22] are warranted in the future.

## Conclusion

Minimum temperature and minimum humidity, at a lag of one month, are positively associated with dengue incidence in the subtropical city of Guangzhou, China. Wind velocity is inversely associated with the dengue incidence of the same month. These findings should be considered in the prediction of future patterns of dengue transmission.

## Competing interests

The authors declare that they have no competing interests.

## Authors' contributions

LL, WZY and QYL conceived and designed the study; HLL, LWT and JMS performed the time-series analysis and drafted the manuscript. All authors contributed to the revision of the manuscript and approved the submitted version of the manuscript.

## Pre-publication history

The pre-publication history for this paper can be accessed here:


